# NPHP and MKS proteins and the ciliary transition zone

**DOI:** 10.1186/2046-2530-1-S1-O13

**Published:** 2012-11-16

**Authors:** BK Yoder

**Affiliations:** 1 University of Alabama at Birmingham, USA

## 

The cilium has emerged as an important signaling and sensory organelle needed for mammalian development and adult tissue homeostasis. It mediates these effects by locating specific receptors, channels, and other signaling factors needed for pathway regulation along the cilium membrane and axoneme. To establish the cilia signaling complement of proteins distinct from that of the cytosol or cell membrane there is a domain called the transition zone (TZ) located at the base that is believed to function as a diffusion barrier, restricting protein entry or exit from the cilium. The structure of the TZ has been well defined through EM analysis, but the proteins required to generate the TZ are only now being identified. Using *C. elegans* models, we have shown that many of the proteins involved in human ciliopathies such as Meckel Gruber syndrome (MKS) and Nephronophthisis (NPHP) grossly localize to the TZ. We are currently defining where these proteins localize on structures within the TZ using higher resolution EM approaches. Further, the NPHP and MKS proteins form two genetically interacting complexes in *C. elegans* that when disrupted result in the loss of structures in the TZ, inappropriate ciliary localization of nonciliary proteins, and severe cilia assembly/positioning defects. Based on the synthetic cilia phenotype observed in all *mks;nphp* double mutants that we generated thus far, we conducted an EMS enhancer screen using *nphp-4* single mutants. The goal of the screen was to generate secondary mutations that would genetically interact with the initial *nphp-4* mutation. Our prediction is that this would yield novel mutations in proteins that function with the MKS or NPHP complexes and would thus provide ideal candidates for novel loci involved in human ciliopathies patients. Using whole genome sequence analysis, we have begun identifying the affected genes. As predicted, three of the alleles were in known MKS genes. Several other mutations in cilia genes have been identified but have not previously been associated with either NPHP or MKS. The role of these genes and the interaction with the NPHP and MKS complexes are being further explored in both *C. elegans* and mouse models. Understanding how the TZ forms and how mutations in TZ proteins disrupt the diffusion barrier will provide important insights into the ciliopathies and how this organelle serves its diverse signaling and sensory functions.

